# Supplementary material for: Characterization of the Bacterial Communities in Cichorium intybus According to Cultivation and Storage Conditions
Source: Microorganisms. 2023 Jun 12;11(6):1560. doi: 10.3390/microorganisms11061560 (PMC10301894; doi:10.3390/microorganisms11061560)
Supplement: Supplementary file 1 [file microorganisms-11-01560-s001.zip › microorganisms-2428626-supplementary.pdf]

Table S1. PCR primers used in this study.

Target bacteria	Target gene	Primer sequence (5'→3')	References
Microbiota	16S rRNA gene V5/V6	F : CMGGATTAGATACCCKGGT<br>R : GGGTTGCGCTCGTTGC	[1]
Acinetobacter lwoffii	*bla*$_{OXA\text{-}134\text{-}like}$	F : CAGGAAGTACAACGCATCCA<br>R : TGCTGGACTTGAGGATCAAA	[2]
Bacillus cereus	*motB*	F : CGCCTCGTTGGATGACG<br>R : GATATACATTCACTTGACTAATACCG	[1]
Enterohemorrhagic *Escherichia coli*	*stx1*	F : CTGGATTTAATGTCGCATAGTG<br>R : AGAACGCCCACTGAGATCATC	[3]
Enteropathogenic *Escherichia coli*	*eaeA*	F : ATGCTTAGTGCTGGTTTAGG<br>R : GCCTTCATCATTTCGCTTTC	[3]
Enterotoxigenic *Escherichia coli*	*stIa*<br>*st*	F : TTTCCCCTCTTTTAGTCAGTCAA<br>R : GCAGGATTACAACACAATTCACAGCAG	[3]
Klebsiella pneumoniae	16S–23S rRNA internal transcribed spacer (ITS)	F : ATTTGAAGAGGTTGCAAACGAT<br>R : CCGAAGATGTTTCACTTCTGATT	[4]
Staphylococcus aureus	*sa442*	F : GTCGGGTACACGATATTCTTCACG<br>R : CTCGTATGACCAGCTTCGGT	[5]
Serratia marcescens	*luxS*	F : TGCCTGGAAAGCGGCGATGG<br>R : CGCCAGCTCGTCGTTGTGGT	[6]

References

1. Yu, Y.-C.; Yum, S.-J.; Jeon, D.-Y.; Jeong, H.-G. Analysis of the microbiota on lettuce (*Lactuca sativa* L.) cultivated in South Korea to identify foodborne pathogens. *J. Microbiol. Biotechnol.* **2018,** *28,* 1318-1331.
2. Kamolvit, W.; Higgins, P.G.; Paterson, D.L.; Seifert, H. Multiplex PCR to detect the genes encoding naturally occurring oxacillinases in *Acinetobacter* spp. *J. Antimicrob. Chemother.* **2014**, *69*, 959-963.
3. Guion, C.E.; Ochoa, T.J.; Walker, C.M.; Barletta, F.; Cleary, T.G. Detection of diarrheagenic *Escherichia coli* by use of melting-curve analysis and real-time multiplex PCR. *J. Clin. Microbiol.* **2008**, *46*, 1752-1757.
4. Martínez-Bastidas, T.; Castro-del Campo, N.; Mena, K.; Castro-del Campo, N.; León-Félix, J.; Gerba, C.; Chaidez, C. Detection of pathogenic micro-organisms on children's hands and toys during play. *J. Appl. Microbiol.* **2014**, *116*, 1668-1675.
5. Grisold, A.J.; Leitner, E.; Mühlbauer, G.; Marth, E.; Kessler, H.H. Detection of methicillin-resistant *Staphylococcus aureus* and simultaneous confirmation by automated nucleic acid extraction and real-time PCR. *J. Clin. Microbiol.* **2002**, *40*, 2392-2397.
6. Joyner, J.; Wanless, D.; Sinigalliano, C.D.; Lipp, E.K. Use of quantitative real-time PCR for direct detection of *Serratia marcescens* in marine and other aquatic environments. *Appl. Environ. Microbiol.* **2014**, *80*, 1679-1683.
